# Stochastic Thermodynamics of Learning Parametric Probabilistic Models

**DOI:** 10.3390/e26020112

**Published:** 2024-01-25

**Authors:** Shervin S. Parsi

**Affiliations:** 1 Physics Program, The Graduate Center, City University of New York, New York, NY 10016, USA; shsparsi@gmail.com; 2 Initiative for the Theoretical Sciences, The Graduate Center, City University of New York, New York, NY 10016, USA

**Keywords:** parameritic generative models, machine learning, thermodynamics of information, entropy production, information theory

## Abstract

We have formulated a family of machine learning problems as the time evolution of parametric probabilistic models (PPMs), inherently rendering a thermodynamic process. Our primary motivation is to leverage the rich toolbox of thermodynamics of information to assess the information-theoretic content of learning a probabilistic model. We first introduce two information-theoretic metrics, memorized information (M-info) and learned information (L-info), which trace the flow of information during the learning process of PPMs. Then, we demonstrate that the accumulation of L-info during the learning process is associated with entropy production, and the parameters serve as a heat reservoir in this process, capturing learned information in the form of M-info.

## 1. Introduction

Starting nearly half a century ago, physicists began to learn that information is a physical entity [1,2,3]. Today, the information-theoretic perspective has significantly impacted various fields of physics, including quantum computing [4], cosmology [5], and thermodynamics [6]. Simultaneously, recent years have witnessed the remarkable success of an algorithmic approach known as machine learning, which is adept at learning information from data. This paper is propelled by a straightforward proposition: if “information is physical”, then the process of learning information must inherently be a physical process.

The concepts of memory, prediction, and information exchange between subsystems have undergone extensive exploration within the realms of thermodynamics of information [6] and stochastic thermodynamics [7]. For instance, Still et al. [8] delved into the thermodynamics of prediction, and the role of information exchange between thermodynamic subsystems has been studied by Sagawa and Ueda [9] and Esposito et al. [10]. This rich toolbox of thermodynamics of information is our main venue to study the physics of the machine learning process, with motivation to assess the information content of the learning process.

The type of machine learning problem we consider in this study encompasses any algorithmic approach that *evolves* a parametric probabilistic model (PPM) or simply the model toward a desirable target distribution through gradient-based loss function minimization. To establish our notation, consider a set of observations denoted by the training dataset *B*, drawn from an unknown target distribution $p^{*}$. The PPM, without lose of generality, can be written as the follows:

(1) $$p_{\theta }\left(X=x\right)=e^{-\phi_{\theta }\left(x\right)}$$

This distribution is parameterized by a set of parameters $\theta \in R^{M}$. The objective of learning is to find a set of parameters such that the samples drawn from the model exhibit a desirable statistical characteristics. In machine learning practice, one constructs the function $\phi_{\theta }\left(x\right)$ with a (deep) neural network and leaves the parameter selection task to an optimizer that minimizes a loss function. Examples include energy-based models [11], Large Language Models (LLMs), Softmax classifiers, and Variational Autoencoders (VAEs) [12].

While the information-theoretic approach to this problem is prevalent in the field [13,14,15,16], it has also faced criticism [17]. Our primary motivation for framing learning in a PPM as a thermodynamic process is to facilitate the assessment of the information content inherent in the learning process. The structure of this paper is as follows. Section 2 briefly discusses prior information-theoretic approaches to the learning problem with PPM and the challenges they encounter. Subsequently, we introduce our own information-theoretic metrics. Finally, Section 3 and Section 6 employ the thermodynamic framework to address these information-theoretic inquiries.

## 2. Information Content of PPMs

Locating information within the parametric model (i.e., the neural network) remains a fundamental question in machine learning [18]. This challenge is central to any information-theoretic perspective on machine learning problems. In a pioneering study, Shwartz-Ziv et al. [19] quantified the internal information within neural networks by estimating the mutual information between inputs and the activities of hidden neurons. Moreover, they employed the information bottleneck theory to interpret the decrease in this mutual information as evidence of data compression during learning. This perspective garnered significant attention in the field [15,16], reinforcing the view of neural networks as an information channel. However, the study encountered critiques [17,20]. A primary problem was that the hidden neurons’ activity in a neural network constitutes a deterministic function of the input. Such determinism inherently possesses a trivial mutual information value, even prior to any learning. The challenge of defining a well-defined and interpretable (Shannon) information metric in deterministic neural networks has prompted the proposition that neural network information processing is geometric in nature [17] (given that inputs are mapped to a latent space of differing dimensions) rather than information-theoretical.

In a distinct research direction, the authors of [21] addressed the significance of assessing the information content of the model’s parameters. In our study, we echo this view, emphasizing that the parameters are the primary carriers of learned information within neural networks. Consequently, any information-theoretic measure of learned information by the model should be grounded in the parameters rather than the deterministic activity of hidden neurons. However, quantifying the information within parameters poses challenges, primarily due to the elusive nature of their distribution [22]. In this section, we introduce two information-theoretic metrics crafted to assess the information content within the learning process of a PPM. This paves the way for computing these quantities within the thermodynamic framework.

To avoid introducing new notation, we also denote *B* as the ground truth random variable associated with the target distribution $p^{*}$ from which the training dataset is sampled. Subsequently, we represent the action of the optimizer as a map between this ground truth random variable and the desired set of parameters after *n* optimization steps:

(2) $$\Theta_{t_{n}}=\Lambda_{n}\left(B\right)$$

The map $\Lambda_{n}$ incorporates the structure of the loss function, the optimization algorithm, and any hyperparameters related to the optimizer’s action. We exclude the initial parameters’ value from this map’s argument under the assumption that as *n* increases, the final set of parameters becomes independent of its initial condition. In information theory terminology, this map corresponds to a *statistic* of the ground truth random variable [23]. Moreover, the outcome of this map defines a model from which the final model-generated sample is sampled: $X_{t_{n}}∼p\left(x|\theta_{t_{n}}\right)$. Considering that the model-generated sample becomes independent of the ground truth random variable given the parameters, we can express the following Markov chain governing the learning process:

(3) $$B\to \Theta_{t_{n}}\to X_{t_{n}}.$$

The data processing inequality (DPI) associated with this Markov chain serves as our framework to define two information-theoretic metrics that gauge the information content of the model:

(4) $$\underset{M\text{-}\mathrm{info}}{\underset{︸}{I_{\Theta ;B}\left(t_{n}\right)}}\geq \underset{L\text{-}\mathrm{info}}{\underset{︸}{I_{X;B}\left(t_{n}\right)}}.$$

We used the notations presented in Table 1. The left-hand side of this inequality quantifies the accumulation of mutual information between the parameters and the training dataset, while the right-hand side characterizes the performance of the generative model, which gauges the accumulation of mutual information between the model’s generated samples and the training dataset. We refer to the former as memorized information (M-info) and the latter as learned information (L-info). We also note that both of these quantities start at zero before the training begins. Thus, their measurements at $t_{n}$ reveal accumulation of information during the learning process.

In the context of the learning problem, the DPI, as referenced in Equation (4), suggests that what is *memorized* is always greater than or equal to what is *learned*. The L-info metric is task-oriented. For example, in the realm of image generation, it quantifies the statistical resemblance between the model’s outputs and the genuine images. In the case of classification tasks, L-info would encapsulate only the pertinent information for label prediction. In contrast, M-info can encompass information not directly pertinent to the current task. For instance, it might capture intricate pixel configurations in an image dataset, which are not crucial for identifying distinct patterns like human faces. The DPI in Equation (4) neatly illustrates the risk of overfitting, which is when a model starts to incorporate extraneous information that does not align with the learning objective. The necessity of constraining the information in a model’s parameters is highlighted in [21], echoing the minimum description length principle [24]. Additionally, studies suggest that the SGD optimizer tends to favor models with minimal information in their parameters [22]. The recent work by the authors of [25] even proposed an upper limit for minimizing parameter information to bolster generalization capabilities. These findings suggest that the learning process seeks to minimize the left-hand side of the DPI inequality while simultaneously maximizing the right-hand side, which measures the model performance. This leads us to an ideal scenario where $I_{\Theta ;B}\left(t_{n}\right)=I_{X;B}\left(t_{n}\right)$, signifying that all memorized information is relevant to the learning task.

We now take one step further in our definition of M-info and L-info. First, the presence of the optimizer map, as referenced in Equation (2) and connecting the ground truth source of the training dataset to the parameters, allows us to simplify M-info as follows:

(5) $$M\text{-}\mathrm{info}:=I_{\Theta ;B}\left(t_{n}\right)=S\left(\Theta_{t_{n}}\right)$$

Thus, the parameters naturally emerge as the model’s *memory*, where its Shannon entropy measures the stored information during the learning process.

Second, we swap *B* for $\Theta_{t_{n}}$ in the definition of L-info at the cost of losing some information:

(6) $$\begin{array}{cc} L\text{-}\mathrm{info}:=I_{X;B}\left(t_{n}\right) & =I_{X;\Lambda_{t_{n}}\left(B\right)}+\epsilon \\ \; & =I_{X;\Theta }\left(t_{n}\right)+\epsilon \end{array}$$

where ϵ is a non-negative number that equals zero only when $\Lambda_{n}$ is a *sufficient statistic* of *B*. For the above expression, the condition of a sufficient statistic can be eased when $\Theta_{t_{n}}$ becomes sufficient only with respect to *X*. In other words, when $\Lambda_{n}$ preserves all information in *B* that is also mutual in $X_{t_{n}}$, i.e., preserving all task-oriented information. Indeed, we are interested in this type of preservative map in machine learning practice. Therefore, for a well-chosen optimizer, we consider $I_{X;\Theta }$ to be a reasonable proxy to L-info, and we use the two interchangeably:

(7) $$L\text{-}\mathrm{info}:=I_{X;\Theta }\left(t_{n}\right)$$

## 3. The Learning Trajectory of a PPM

The time evolution of the PPM is the first clue to frame the learning process as a thermodynamic process. To illustrate this, consider a discretized time interval $\left[0,t_{n}\right]$, which represents the time needed for *n* optimization steps of the parameters. During this time, the optimizer algorithm draws a sequence of independent and identically distributed samples from the training dataset. We denote this sequence by $b_{n}:=\left\{b_{t_{1}},b_{t_{2}},\cdots ,b_{t_{n}}\right\}$ and refer to it as the “input trajectory”. Then, the outcome of the optimization defines a sequence of parameters called the “parameters’ trajectory”: $\theta_{n}:=\left\{\theta_{0},\theta_{t_{1}},\theta_{t_{2}},\cdots ,\theta_{t_{n}}\right\}$. Each realization of parameters defines a specific PPM. Consequently, the parameters’ trajectory produces a sequence of PPMs:

(8) $$T:=\{p\left(X|\theta_{0}\right),\;p\left(X|\theta_{t_{1}}\right),\;p\left(X|\theta_{t_{2}}\right),\cdots ,\;p\left(X|\theta_{t_{n}}\right)\}$$

**Figure 1:**
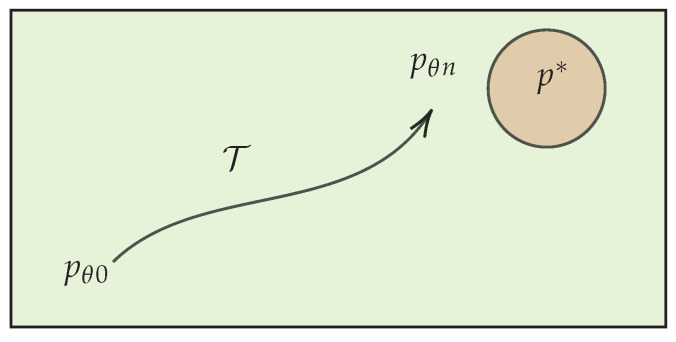
The learning trajectory T depicts the thermodynamic process that takes the initial model state to the final state. The green area shows the space of family of distribution accessible to the PPM. The red area considers the possibility that the target distribution $p^{*}$ is not in this family.

We refer to this sequence as the *learning trajectory*, depicted in Figure 1. On the other hand, a thermodynamic process can be constructed solely from the time evolution of a distribution [26]. Therefore, we see T as a thermodynamic process. The physics of this process is encoded in the transition rates governing the master equation of this time evolution. Finding the transition rate associated to learning a PPM is our main task in this section.

### 3.1. The Model Subsystem

We refer to the subsystem that goes under the thermodynamic process T as *the model subsystem*. This subsystem has *X* degrees of freedom, and its microscopic states’ realization along the learning trajectory represent model-generated samples at each time step. We denote the stochastic trajectory of the model subsystem (model-generated samples) by $x_{n}:=\left\{x_{t_{1}},x_{t_{2}},\cdots ,x_{t_{n}}\right\}$. To avoid confusion with our notation, we consider the probability functions $p(x_{t_{i}}|\theta_{t_{i}})$ and $p(x_{t_{i-1}}|\theta_{t_{i}})$, which represent the probability of observing $x_{t_{i}}\in x_{n}$ and $x_{t_{i-1}}\in x_{n}$ at time $t=t_{i}$, respectively. Here, the time index of θ aligns with the time index of the PPM (i.e., $p_{t_{i}}\left(X|\theta_{t_{i}}\right)\equiv p\left(X|\theta_{t_{i}}\right)$) because the PPM is fully defined upon observing the parameters. In contrast, the time index on *x* denotes a specific observation within $x_{n}$. To simplify our notation, the absence of a time index on *x* denotes a generic realization of the random variable *X*, and we write $p(x|\theta_{t_{i}})$ instead of $p(X|\theta_{t_{i}})$.

### 3.2. The Parameter Subsystem

The parameters of the neural network at each step of optimization represent realization of the parameter subsystem with Θ degrees of freedom and the stochastic trajectory $\theta_{n}$. The statistical state of the parameter subsystem is given with the marginal $p(\theta_{t_{i}})$ at time step $t=t_{i}$. This marginal state represents the statistic of all possible outcomes of training a PPM on a specific learning objective. We can think of training an ensemble of computers on the same machine learning task. This allows us to think about the time evolution of the marginal $p(\theta_{t_{i}})$ and the joint distribution $p\left(x|\theta_{t_{i}}\right)p\left(\theta_{t_{i}}\right)$ during the learning process. We refer to this view as the *ensemble view* of the learning process. In practice, however, we train the PPM only once, and we do not have access to the marginal $p(\theta_{t_{i}})$. Thus, our model-generated samples are conditioned on specific observations of parameters $\theta_{t_{i}}∼\Theta_{t_{i}}$. This defines the *conditional view* of the learning process, which is fully described by the learning trajectory of the PPM.

In machine learning practice, it is desirable for a training process to exhibit a robust outcome, regardless of who is running the code. One way to achieve this is by imposing a *low variance condition* on the statistics of the parameters across the ensemble of all learning trials. This condition asserts that the parameters’ trajectory across the ensemble is confined to a small region $D_{n}\subset R^{M}$. As *n* grows larger, this region shrinks and becomes associated with the area surrounding the target distribution as depicted in Figure 1. Under this condition, we can express

(9) $$<f\left(\theta \right){>}_{p(\theta_{n})}\approx f\left(\theta^{*}\right),\;\;\forall \theta^{*}\in D_{n}$$

The above approximation becomes exact when $p(\theta_{n})$ assumes the form of a Dirac delta function, indicating a zero-variance condition in the parameters’ dynamics.

The low-variance condition proves invaluable when computing the information-theoretic measurements introduced in Section 2. This is because the computation of the M-info $I_{B;\Theta }$ and L-info $I_{X;\Theta }$ necessitates averaging over the parameters’ distribution. However, since we typically train our model just once, we lack direct access to the parameters’ distribution throughout the learning trajectory. To overcome this challenge, we introduce the partially averaged L-info:

(10) $$I_{X:\Theta }\left(\theta_{t}\right)=\int dx\;p\left(x|\theta_{t}\right)\ln\frac{p(x|\theta_{t})}{p_{t}\left(x\right)}$$

Subsequently, under the low-variance condition of the parameter subsystem, we can measure the partially averaged L-info as a proxy for the L-info, where $I_{X:\Theta }\left(t\right)=<I_{X:\Theta }\left(\theta \right){>}_{p_{t}\left(\theta \right)}\approx I_{X:\Theta }\left(\theta_{t}\right)$. In Section 6, we will delve deeper into the evidence supporting the low-variance condition of the subsystem Θ.

We refer to the joint (X,Θ) as the *learning system*, which embodies the thermodynamic process of learning a PPM. In this section, we will demonstrate that the thermodynamic exchange between the model subsystem and parameter subsystem is the primary source for producing M-info and L-info during the learning process. Before delving deeper, we establish two interconnected assumptions about the parameter subsystem: (1) The PPM is over-parameterized; specifically, the subsystem Θ has a much higher dimension compared with the subsystem *X*. (2) The parameter subsystem evolves in a quasi-static fashion (slow dynamics).

The foundation for these assumptions in machine learning is clear. Training over-parameterized models represents a pivotal achievement of machine learning algorithms, and the slow dynamics (often termed as lazy dynamics) of these over-parametrized models are well documented [27,28]. These characteristics underscore the significant role of the parameter subsystem in the learning process, akin to that of a heat reservoir. Over-parameterization implies a higher heat capacity for this subsystem compared with the model subsystem. Additionally, the quasi-dynamics align with the behavior of an ideal heat reservoir, which does not contribute to entropy production [29]. The role of the parameter subsystem as a reservoir aligns with the assumption of a low-variance condition for this subsystem. This is because we expect the stochastic dynamics of the reservoir to be low variance across the ensemble of all trials.

In this study, we attribute the role of an ideal heat reservoir to the parameter subsystem with an inverse temperature $\beta^{-1}=1$. In Section 6, we delve deeper into the rationale behind this assumption by examining the stochastic dynamics of parameters under a vanilla stochastic gradient descent optimizer and highlighting potential limitations of this assumption.

### 3.3. Lagged Bipartite Dynamics

We want to emphasize that the dynamics of subsystem *X* are not a mere conjecture or an arbitrary component in this study. Rather, it is an integral part of training a generative PPM. These dynamics are inherent in the optimizer action, necessitating a fresh set of model-generated samples to compute the loss function or its gradients after each parameter update. For instance, in the context of EBM, a Langevin Monte Carlo (LMC) sampler can be employed to generate new samples from the model [30]. The computational cost of producing a fresh set of model-generated samples introduces a time delay in the parameter dynamics. For instance, when using an LMC sampler, the number of Monte Carlo steps dictates this lag time. Conversely, in the case of a language model, since the computation of the loss function relies on inferring subsequent tokens, the inference latency signifies the time delay.

We denote the lag time parameters as τ. Here, the model subsystem evolves on the timescale δt, while the parameter subsystem evolves on the timescale α=τδt. In the thermodynamic context, this parameter represents the *relaxation time* of the subsystem *X* under the fixed microscopic state of the subsystem Θ. Conceptually, parameter τ acts as a complexity metric, quantifying the computational resources required for each parameter optimization step. Moreover, the dynamics of the joint (X,Θ) exhibit a bipartite property. This implies that simultaneous transitions in the states of *X* and Θ are not allowed, given that the observation of a new set of model-generated samples occurs only after a parameter update.

The lagged bipartite dynamics described above can be represented using two time resolutions: δt and α. In the finer time resolution of δt, the Markov chain within the time interval $\left[t_{i},t_{i+1}\right]$ is as follows:

(11) $$\begin{array}{c} \left(x_{t_{i}},\theta_{t_{i}}\right)\to \left(x_{t_{i}},\theta_{t_{i+1}}\right)\to \left(x_{t_{i}+\delta t},\theta_{t_{i+1}}\right)\;\cdots \;\to \left(x_{t_{i}+\tau \delta t},\theta_{t_{i+1}}\right)\equiv \left(x_{t_{i+1}},\theta_{t_{i+1}}\right). \end{array}$$

We can also analyze this dynamics at a coarser time resolution of α. Within the interval $\left[t_{0},t_{n}\right]$, the Markov Chain appears as

(12) $$\begin{array}{c} \left(x_{0},\theta_{0}\right)⤏\left(x_{t_{1}},\theta_{t_{1}}\right)\;\cdots \;\left(x_{t_{n-1}},\theta_{t_{n-1}}\right)⤏\left(x_{t_{n}},\theta_{t_{n}}\right). \end{array}$$

**Figure 2:**
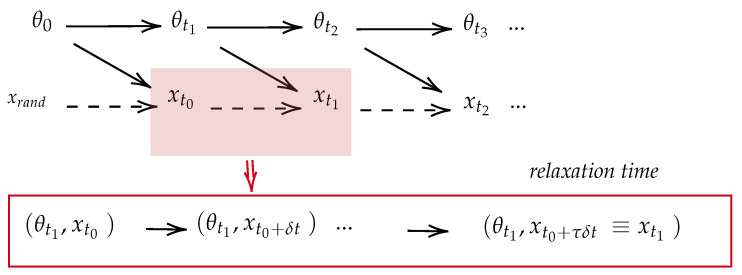
This figure shows the Bayesian network for the joint trajectory probability $P[x_{n},\theta_{n}]$ based on the lagged bipartite dynamics.

In the above Markov chain, the dashed arrows remind us of the *ignorance* of the intermediate steps in the high resolution picture (Equation (11)). Figure 2 illustrates the lagged bipartite dynamics of the learning system. An important observation is that the learning trajectory T, as defined in Equation (8), is written in the low-resolution picture. Therefore, studying the learning trajectory means studying the dynamics of the system (X,Θ) in the low- resolution picture.

### 3.4. Trajectory Probabilities

To set the stage for the application of the fluctuation theorem (FT) to learning a PPM, we define the trajectory probability of the joint $\left(x_{n},\theta_{n}\right)$ as the probability of observing the trajectory of model-generated samples and parameters during the learning process:

(13) $$\begin{array}{c} P\left[x_{n},\theta_{n}\right]:=p\left(x_{0},x_{t_{1}},\cdots ,x_{t_{n}},\theta_{0},\theta_{t_{1}},\cdots ,\theta_{t_{n}}\right) \end{array}$$

Additionally, we can consider the time reversal of the model’s trajectory and parameters’ trajectory as ${\tilde{x}}_{n}:=\left\{x_{t_{n}},x_{t_{n-1}},\cdots ,x_{t_{1}}\right\}$ and ${\tilde{\theta }}_{n}:=\left\{\theta_{t_{n}},\theta_{t_{n-1}},\cdots ,\theta_{t_{1}}\right\}$, respectively. Then, the probability of observing the backward trajectory is denoted by $P[{\tilde{x}}_{n},{\tilde{\theta }}_{n}]$.

Here, $P[x_{n},\theta_{n}]$ represents the trajectory probability of the learning system in the ensemble view. In practice, however, we typically train our model only once, and we often lack access to the parameters’ distribution. Therefore, our model is conditioned on the observation of a specific parameters’ trajectory $\theta_{n}$. This defines the trajectory probability in the conditional view:

(14) $$P\left[x_{n}|\theta_{n}\right]:=\frac{P[x_{n},\theta_{n}]}{P[\theta_{n}]}$$

where

(15) $$P\left[\theta_{n}\right]=p\left(\theta_{0},\theta_{t_{1}},\cdots ,\theta_{t_{n}}\right).$$

Similarly, the backward conditional trajectory probability is the probability of observing the time reversal model’s trajectory, conditioned on the observation of the time reversal parameters’ trajectory $P\left[{\tilde{x}}_{n}|{\tilde{\theta }}_{n}\right]=\frac{P[{\tilde{x}}_{n},{\tilde{\theta }}_{n}]}{P[{\tilde{\theta }}_{n}]}$.

We now use the Markov property of the Markov chain in Equation (12) to decompose the conditional trajectory probability and the marginal trajectory probability, respectively, as follows:

(16) $$\begin{array}{cc} P[x_{n}|\theta_{n}] & =p\left(x_{t_{n}}|x_{t_{n-1}},\theta_{t_{n}}\right)\cdots p\left(x_{t_{1}}|x_{0},\theta_{t_{1}}\right)p\left(x_{0}|\theta_{0}\right),\\ P[\theta_{n}] & =p\left(\theta_{t_{n}}|\theta_{t_{n-1}}\right)\cdots p\left(\theta_{t_{1}}|\theta_{t_{0}}\right)p\left(\theta_{0}\right), \end{array}$$

where expressions such as $p(x_{t_{n}}|x_{t_{n-1}},\theta_{t_{n}})$ and $p(\theta_{t_{n}}|\theta_{t_{n-1}})$ represent the transition probabilities that determine the probability of moving from one microscopic state to another. Additionally, we define two probability trajectories conditioned on the initial conditions, which will be used later in the formulation of the FT:

(17) $$\begin{array}{cc} P[\left(x_{n}|\theta_{n}\right)|\left(x_{0}|\theta_{0}\right)] & :=P\left[\left(x_{n}|\theta_{n}\right)\right]/p\left(x_{0}|\theta_{0}\right),\\ P[\theta_{n}|\theta_{0}] & :=P\left[\theta_{n}|\theta_{0}\right]/p\left(\theta_{0}\right). \end{array}$$

### 3.5. Local Detailed Balance (LDB) for Learning PPMs

The transition probabilities, represented in Equation (16), capture the physics of the learning problem. When considering a Markov property (i.e., memoryless process) for time evolution of the model subsystem, the transition rate for this subsystem is reduced to the PPM:

(18) $$p\left(x_{t_{i}}|x_{t_{i-1}},\theta_{t_{i}}\right)=p\left(x_{t_{i}}|\theta_{t_{i}}\right).$$

The above expression suggests that the transition rate between two microscopic states $x_{t_{i-1}}$ and $x_{t_{i}}$ under the fixed $\theta_{t_{i}}$ is equivalent to the probability of observing $x_{t_{i}}$ by the PPM itself at $t=t_{i}$. To reiterate, this is the Markov property that suggests the elements inside $x_{n}$ are independently and freshly drawn from the PPM specified with given parameters along the learning trajectory T. This is especially true when τ>>1. We can generalize this observation for the backward transition probability $p(x_{t_{i-1}}|x_{t_{i}},\theta_{t_{i}})$, which represents the probability of the backward transition $\left(x_{t_{i}},\theta_{t_{i}}\right)⤏\left(x_{t_{i-1}},\theta_{t_{i}}\right)$ under a fixed $\theta_{i}$, as follows:

(19) $$p\left(x_{t_{i-1}}|x_{t_{i}},\theta_{t_{i}}\right)=p\left(x_{t_{i-1}}|\theta_{t_{i}}\right).$$

The above expression tells us that the probability of backward transition is equivalent to the probability of observing the sample generated at $t=t_{i-1}$ in $x_{n}$ with the PPM at time $t=t_{i}$.

Finally, we write the log ratio of the forward and backward transitions:

(20) $$\ln\frac{p(x_{t_{i}}|x_{t_{i-1}},\theta_{t_{i}})}{p(x_{t_{i-1}}|x_{t_{i}},\theta_{t_{i}})}=\ln\frac{p(x_{t_{i}}|\theta_{t_{i}})}{p(x_{t_{i-1}}|\theta_{t_{i}})}=-\left(\phi_{\theta_{t_{i}}}\left(x_{t_{i}}\right)-\phi_{\theta_{t_{i}}}\left(x_{t_{i-1}}\right)\right),$$

where the second equality is due to Equation (1). The above expression resembles the celebrated local detailed balance (LDB) [31], which relates the log ratio of the forward and backward transition probabilities to the difference in potential energy of the initial and final states in the transition. The heat reservoir that supports the legitimacy of the above LBD expression for the learning PPM is the parameter subsystem, whose temperature has been set to one, as we will discuss in more detail in Section 6. We emphasize that the above LBD emerged naturally under assumption of the Markov property and a relaxation time for learning a generic generative PPM. It is also important to note that the above LBD is only valid in the low-resolution picture.

The LBD relation, presented in Equation (20), has a profound consequence. This allows us to write the forward conditional probability trajectory $P[x_{n}|\theta_{n}]$ and the backward conditional probability trajectory $P[{\tilde{x}}_{n}|{\tilde{\theta }}_{n}]$ solely based on the series of PPMs in the learning trajectory T:

(21) $$\begin{array}{cc} P[x_{n}|\theta_{n}] & =p\left(x_{t_{n}}|\theta_{t_{n}}\right)\;\cdots \;p\left(x_{t_{1}}|\theta_{t_{1}}\right)\;p\left(x_{0}|\theta_{0}\right)\\ P[{\tilde{x}}_{n}|{\tilde{\theta }}_{n}] & =p\left(x_{0}|\theta_{t_{1}}\right)\;\cdots \;p\left(x_{t_{n-1}}|\theta_{t_{n}}\right)\;p\left(x_{t_{n}}|\theta_{t_{n}}\right) \end{array}$$

This is significant because it renders the application of the FT framework to the learning PPMs practically, as we have access to elements of the learning trajectory during the learning process.

## 4. L-Info and Entropy Production

The version of the fluctuation theorem we are about to apply to the learning PPMs is known as the detailed fluctuation theorem (DFT) [32]. We also note that the machinery we are about to present for measuring the information flow in PPMs was developed to study the information exchange between thermodynamic subsystems [9]. The novelty here lies merely in the application of this machinery to the learning process of a PPM. In this section, we extensively use the notations presented in Table 1. Also, note that the temperature of the parametric reservoir is set to one. Applying the DFT in the conditional view (i.e., the conditional forward and backward trajectories defined in Equation (21)) results in

(22) $$\begin{array}{cc} \sigma_{x_{n}|\theta_{n}} & =\ln\frac{P[x_{n}|\theta_{n}]}{P[{\tilde{x}}_{n}|{\tilde{\theta }}_{n}]}\\ \; & =\ln\frac{P[\left(x_{n}|\theta_{n}\right)|\left(x_{0}|\theta_{0}\right)]}{P[\left({\tilde{x}}_{n}|{\tilde{\theta }}_{n}\right)|\left(x_{t_{n}}|\theta_{t_{n}}\right)]}+\ln\frac{p(x_{0}|\theta_{0})}{p(x_{t_{n}}|\theta_{t_{n}})}\\ \; & =-q_{x_{n}}\left(\theta_{n}\right)+s\left[p\left(x_{t_{n}}|\theta_{t_{n}}\right)\right]-s\left[p\left(x_{t_{0}}|\theta_{t_{0}}\right)\right] \end{array}$$

The first line is due to the DFT, which defines the stochastic Entropy Production (EP) to be the logarithm of the ratio of the forward and backward trajectory probabilities. The second line is due to the decomposition presented in Equation (17). Finally, the third line is the consequence of the LDB relation in Equation (20), and the definition of the stochastic heat flow $q_{x_{n}}\left(\theta_{n}\right)$ is the change in the energy of the subsystem *X* due to alterations in its microscopic state configuration:

(23) $$q_{x_{n}}\left(\theta_{n}\right):=-\ln\frac{P[\left(x_{n}|\theta_{n}\right)|\left(x_{0}|\theta_{0}\right)]}{P[\left({\tilde{x}}_{n}|{\tilde{\theta }}_{n}\right)|\left(x_{t_{n}}|\theta_{t_{n}}\right)]}=\sum_{i=1}^{n}\;\phi_{\theta_{t_{i}}}\left(x_{i}\right)-\phi_{\theta_{t_{i}}}\left(x_{i-1}\right).$$

Note that our convention defines $q_{x_{n}}>0$ as the heat absorbed by the subsystem *X*.

The second law arises from averaging Equation (22) over the forward trajectory distribution $P[x_{n}|\theta_{n}]$ and recalling the nonnegativity property of the Kl divergence to establish the nonnegativity of the averaged EP; $\Sigma_{X|\Theta }\left(\theta_{n}\right):=<\ln\frac{P_{F}\left[x_{n}|\theta_{n}\right]}{P_{B}\left[{\tilde{x}}_{n}|{\tilde{\theta }}_{n}\right]}{>}_{P_{F}\left[x_{n}|\theta_{n}\right]}\;\geq 0$. We note that this still a partially averaged EP, conditioned on the stochastic trajectory of the parameters. This is indeed the consequence of working in the conditional view.

Motivated to compute the L-info, in the next step, we rearrange Equation (22) as follows:

(24) $$I\left[x_{t_{n}}:\theta_{t_{n}}\right]-I\left[x_{0}:\theta_{0}\right]=-q_{x_{n}}\left(\theta_{n}\right)+s\left[p\left(x_{t_{n}}\right)\right]-s\left[p\left(x_{t_{0}}\right)\right]-\sigma_{x_{n}|\theta_{n}},$$

where $I\left[x_{t_{n}}:\theta_{t_{n}}\right]:=s\left[p\left(x_{t_{n}}\right)\right]-s\left[p\left(x_{t_{n}}|\theta_{t_{n}}\right)\right]$ is the mutual content (or stochastic mutual information) at $t=t_{n}$. We now arrive at the partially averaged L-info (Equation (10)) by averaging Equation (24) over $P[x_{n}|\theta_{n}]$:

(25) $$\begin{array}{cc} I_{X;\Theta }\left(\theta_{t_{n}}\right)-I_{X;\Theta }\left(\theta_{0}\right) & =-Q_{X}\left(\theta_{n}\right)+\left(S_{X}\left(\theta_{t_{n}}\right)-S_{X}\left(\theta_{0}\right)\right)-\Sigma_{X|\Theta }\left(\theta_{n}\right)\\ \; & =\Sigma_{X}\left(\theta_{n}\right)-\Sigma_{X|\Theta }\left(\theta_{n}\right). \end{array}$$

This defines a series of Partially Averaged (PA) quantities:

$$\begin{array}{ccc} Q_{X}\left(\theta_{n}\right) & :=\sum_{i=1}^{n}\;<\phi_{\theta_{t_{i}}}\left(x\right){>}_{p(x|\theta_{t_{i}})}-<\phi_{\theta_{t_{i}}}\left(x\right){>}_{p(x|\theta_{t_{i-1}})} & \left(\mathrm{PA Heat flow}\right)\\ S_{X|\Theta }\left(\theta_{t_{i}}\right) & :=<-log\left(\;p\left(x|\theta_{t_{i}}\right)\;\right){>}_{p(x|\theta_{t_{i}})} & \left(\mathrm{PA Conditinal Entropy}\right)\\ S_{X}\left(\theta_{t_{i}}\right) & :=<-log\left(\;p\left(x\right)\;\right){>}_{p(x|\theta_{t_{i}})} & \left(\mathrm{PA Marginal Entropy}\right)\\ \Sigma_{X}\left(\theta_{n}\right) & :=\left(S_{X}\left(\theta_{t_{n}}\right)-S_{X}\left(\theta_{0}\right)\right)-Q_{X}\left(\theta_{n}\right) & \left(\mathrm{PA Marginal EP}\right) \end{array}$$

We note that all PA quantities are conditioned on the parameters’ trajectory (i.e., the choice of $\theta_{n}$ from the ensemble). This is a direct consequence of working in the conditional view. However, this also signifies that all thermodynamic quantities mentioned above are computable in the practice of machine learning, as they only require access to the time evolution of one PPM. Fortunately, thanks to the low-variance condition in Equation (9), we can use the partially averaged L-info as a proxy to the L-info.

Equation (25) equates the L-info to the difference between the marginal EP, and the conditional EP. We refer to this difference as the *ignorance* EP:

(26) $$\Sigma_{ign}\left(\theta_{n}\right):=\Sigma_{X}\left(\theta_{n}\right)-\Sigma_{X|\Theta }\left(\theta_{n}\right)$$

It is important to note that both the marginal EP and the conditional EP measure the EP of the same process, which is the time evolution of the subsystem *X*. However, the conditional EP measures this quantity with a lower time resolution of α, which is conditioned on the parameter’s subsystem. On the other hand, the marginal EP measures this quantity with a higher time resolution of δt, including the relaxation time of the subsystem *X* between each parameter’s update. Therefore, the term “ignorance” refers to ignorance of the full dynamic of *X*, and the origin of the L-info is the EP between each consecutive parameter’s update (i.e., the EP of generating fresh samples represented with the Markov chain in Equation (11)).

## 5. M-Info and the Parameter Subystem

We can also apply the DFT to subsystem Θ:

(27) $$\begin{array}{cc} \sigma_{\theta_{n}} & =\log\frac{P[\theta_{n}]}{P[\tilde{\theta_{n}}]}\\ \; & =-q_{\theta_{n}}+s\left[p\left(\theta_{t_{n}}\right)\right]-s\left[p\left(\theta_{t_{0}}\right)\right]. \end{array}$$

In the above expression, the second line is due to the decomposition in Equation (17), and the definition of the stochastic heat flow for the parameter subsystem is $q_{\theta_{n}}:=\log P[\theta_{n}|\theta_{0}]/P[\tilde{\theta_{n}}|\theta_{t_{n}}]$.

Under the assumption that the subsystem Θ evolves quasi-statically, the EP of this subsystem is zero, as expected for an ideal reservoir. This results in $q_{\theta_{n}}=\Delta_{t_{n}}s\left[p\left(\theta_{t}\right)\right]$. Furthermore, in the closed system of (X,Θ), the heat flow of the subsystem *X* must be provided with an inverse flow of the subsystem Θ (i.e., $q_{x_{n}}\left(\theta_{n}\right)=-q_{\theta_{n}}$). Thus, we arrive at the stochastic version of the Clausius relation for the heat reservoir:

(28) $$\Delta_{t_{n}}s\left[p\left(\theta_{t}\right)\right]=-q_{x_{n}}\left(\theta_{n}\right)$$

This relation states that the heat dissipation in subsystem *X* ($q_{x_{n}}\left(\theta_{n}\right)<0$) is compensated by an increase in the parameter subsystem entropy. We recall the definition of M-info from Equation (5) as the entropy of the subsystem Θ. Since heat dissipation is a source of L-info accumulation (see Equation (25)), the above Clausius relation states that this information is stored in the parameters by increasing the entropy of this subsystem (i.e., the M-info), confirming the role of parameters as the memory space of the PPM.

We can also take the ensemble average of Equation (28) (i.e., averaging over $P[x_{n},\theta_{n}]$):

(29) $$\Delta_{t_{n}}S\left[\Theta_{t}\right]=-Q_{X}\left(t_{n}\right),$$

where $Q_{X}\left(t_{n}\right):=\sum_{x_{n},\theta_{n}}P\left[x_{n},\theta_{n}\right]\;q_{x_{n}}\left(\theta_{n}\right)=\sum_{\theta_{n}}P\left[\theta_{n}\right]\;Q_{X}\left(\theta_{n}\right)$ is the fully averaged dissipated heat from the subsystem *X*. However, under the low-variance condition of learning (Equation (9)), we expect $Q_{X}\left(\theta_{n}\right)$ to be independent of a specific parameters’ trajectory from the ensemble. Thus, we can write $Q_{X}\left(t_{n}\right)\approx Q_{X}\left(\theta_{n}\right)$.

### The Ideal Learning Process

The learning objective necessitates an increase in L-info to enhance the model’s performance while simultaneously reducing the M-info to minimize the generalization error and prevent overfitting. As previously mentioned in Section 2, the ideal scenario is achieved when all the stored information in the parameters (M-info) matches the task-oriented information learned by the model (L-info). Now that we have studied the machinery for computing these two information-theoretic quantities through the computation of entropy production, we can formally examine this optimal learning condition.

Maximizing the L-info, as described in Equation (25), is equivalent to maximizing the marginal EP while minimizing the conditional EP. Given that the conditional EP is always nonnegative, the “ideal” scenario would involve achieving a conditional EP of zero (i.e., $\Sigma_{X|\Theta }\left(t_{n}\right)=0$). This condition can be realized through a quasi-static time evolution of the PPM occurring on the lower-resolution timescale α, presented in the Markov chain in Equation (12). In the context of generative models, this condition is akin to achieving perfect sampling. Under these circumstances, all EP of the subsystem *X* transforms into L-info, resulting in $\Delta_{t_{n}}I_{X;\Theta }\left(\theta_{t}\right)=\Sigma_{X}\left(t_{n}\right)$.

Thermodynamically, the condition of quasi-static time evolution of the PPM (and consequently the zero conditional EP) can be realized by having a large relaxation parameter τ≫1, which allows the model to reach equilibrium after each optimization step. However, a high relaxation parameter comes at the cost of requiring more computational resources and longer computation times. This introduces a fundamental trade-off between the time required to run a learning process and its efficiency, a concept central to thermodynamics and reminiscent of the Carnot cycle, representing an ideal engine that requires an infinite operation time.

## 6. The Parameters’ Reservoir

In the formulation of the previous sections, we make the assumption that the subsystem Θ behaves as an ideal reservoir. In this section, we delve deeper into the premises of this assumption by studying the dynamics of the parameter subsystem. To facilitate our formulation, we adapt a negative log-likelihood as a fairly general form for the loss function,

(30) $$\ell \left(b_{t},\theta \right):=-\frac{1}{|b_{t}|}\sum_{x\in b_{t}}\log\left(p_{\theta }\left(x\right)\right)=\phi_{\theta }\left(b_{t}\right).$$

Here, the loss function is computed according to the empirical average of a random mini-batch $b_{t}\in B$ drawn from the training dataset at time step *t*. The last equality is due to the PPM defined in Equation (1) and $\phi_{\theta }\left(b_{t}\right):=\frac{1}{|b_{t}|}\sum_{x\in b_{t}}\phi_{\theta }\left(x\right)$, where the notation |·| shows the size of a set. We also use a vanilla stochastic gradient descent (SGD) optimizer with the learning rate *r* to take gradient steps iteratively for *n* steps in the direction of loss function minimization:

(31) $$\theta_{t+1}=\theta_{t}-r\nabla_{\theta }\phi_{\theta }\left(b_{t}\right){|}_{\theta =\theta_{t}}$$

To render the dynamics of parameters in the form of a conventional overdamped Langevin dynamic, we introduce the following conservative potential, defined by the entire training dataset *B*:

(32) $$U_{B}\left(\theta \right):=\frac{1}{\left|B\right|}\sum_{x\in B}\phi_{\theta }\left(x\right).$$

The negative gradient of this potential gives rise to a deterministic vector force. Additionally, we define the fluctuation term, which represents the source of random forces due to selection of a mini-batch at time step $t_{n}$:

$$\begin{array}{cc} \eta (t_{n}) & :=-\nabla_{\theta }\;\phi_{\theta_{t}}\left(b_{t_{n}}\right)+\nabla_{\theta }\;U_{B}\left(\theta_{t_{n}}\right). \end{array}$$

We now reformulate the SGD optimizer in Equation (31) in the guise of overdamped Langevin dynamics, dividing it by the parameters’ update timescale α to convert the learning protocol into a dynamic over time:

(33) $$\begin{array}{c} \frac{\theta_{t_{n+1}}-\theta_{t_{n}}}{\alpha }=-\mu \nabla_{\theta }U_{B}\left(\theta_{t_{n}}\right)\;+\mu \;\eta \left(t_{n}\right), \end{array}$$

where μ:=r/α is known as the mobility constant in the context of Brownian motion.

We note that Equation (33) is merely a rearrangement of the standard SGD. For us to interpret it as a Langevin equation, the term $\eta (t_{n})$ must represent a stationary stochastic process to serve as the *noise* term in the Langevin equation. To demonstrate this property of $\eta (t_{n})$, we must examine the characteristic of its time correlation function (TCF) [33]: $C_{i,j}\left(t,t-t^{\prime }\right):=\delta_{i,j}<\eta_{i}\left(t\right)\eta_{j}\left(t^{\prime }\right)>$, where the indices i,j represent different components of the vector θ and $\delta_{i,j}$ is the Kronecker delta.

If the fluctuation term η satisfies the condition of the white noise (uncorrelated stationary random process), and assuming that Equation (33) describes a motion akin to Brownian motion, then we can apply the fluctuation-dissipation theorem to write

(34) $$<\eta_{i}\left(t\right)\eta_{j}\left(t^{\prime }\right)>=\frac{2k_{B}T}{\mu }\delta \left(t-t^{\prime }\right)\delta_{i,j}$$

Here, $\delta (t-t^{\prime })$ is a delta Dirac, $k_{B}$ stands for the Boltzmann constant, and the constant *T* symbolizes the temperature. To render our framework as unitless, we treat the product of the Boltzmann factor and temperature as dimensionless. Moreover, regardless of the noise width, we set T=1, and henceforth it will not appear in our formulation. This is possible by adjusting the Boltzmann factor according to the noise width (i.e., $k_{B}=\mu <\eta_{i}\left(t\right)\eta_{i}\left(t\right)>/2$).

**Figure 3:**
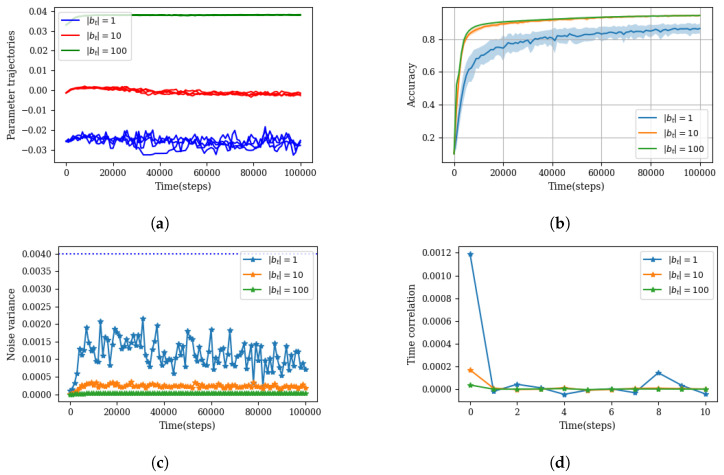
This experiment contrasts the parameter dynamics with three different mini-batch sizes: $|b_{t}|=1$, $|b_{t}|=10$ and $|b_{t}|=100$. The model under consideration is a four-layer feedforward neural network with a uniform width of 200 neurons. It was trained on the MNIST classification task using a vanilla SGD optimizer. The experiment was replicated over 50 trials to generate an ensemble of parameters. (**a**) One random parameter from the model’s last layer was chosen for each batch size scenario, and four of its dynamic realizations are depicted. (**b**) Illustration of both the average accuracy (solid line) and the variance in accuracy within the ensemble (shaded area), emphasizing the low-variance condition, which asserts that macroscopic quantities such as accuracy have low variance statistics across the ensemble. (**c**) The noise variance averaged over all parameters (i.e., $\frac{1}{\dim(\theta )}\sum_{i=0}^{\dim(\theta )}C_{i,i}\left(t,0\right)$) for each mini-batch size scenario, underscoring the stationary nature of η. This part also highlights the role of the mini-batch size in determining the noise width (i.e., the temperature of the environment). The horizontal dashed line indicates the maximum absolute value observed from $\nabla_{\theta }\;U_{B}\left(\theta_{t_{n}}\right)$, serving as a reference point for the magnitude of the noise. (**d**) The autocorrelation of the term η averaged over all parameters. For instance, computing this quantity at step 1000 reads as follows: $\frac{1}{\dim(\theta )}\sum_{i=0}^{\dim(\theta )}C_{i,i}\left(t=1000,t^{\prime }-t\right)$. The rapid decline in autocorrelation with time lag indicates the white noise characteristic of η.

We still need to investigate if the fluctuation term indeed describes an uncorrelated stationary random process, as presented in Equation (34). To this end, we conducted an experiment by training an ensemble of 50 models for the classification of the MNIST dataset. To induce different levels of stochastic behavior (i.e., different “temperatures”), we considered three different mini-batch sizes. A smaller mini-batch size leads to a bigger deviation in the fluctuation term, consequently amplifying the influence of random forces. The results are presented in Figure 3.

The plot in Figure 3c represents the TCF function at no time lag $t=t^{\prime }$ (i.e., a variance of η(t)) as a function of time. The constant value of variance suggests the stationary property of η(t). Moreover, Figure 3d illustrates the autocorrelation of η(t) at different time lags, indicating the white noise characteristic for this term.

However, it would be naive to draw a generic conclusion regarding the nature of the fluctuation term as an uncorrelated stationary random process solely based on a simple experiment. Indeed, research has demonstrated that the noise term can be influenced by the Hessian matrix of the loss function [34]. This observation aligns with our definition of the fluctuation term presented in Equation (33), where η is defined in relation to the gradient of the loss itself. Consequently, as the optimizer explores the landscape of the loss function, the characteristics of the fluctuation term η can vary. We can grasp this concept in the context of Brownian motion by envisioning a Brownian particle transitioning from one medium to another, each with distinct characteristics. This implies that there could be intervals during training where η stays independent of the loss function and exhibits a stationary behavior.

Moreover, we overlooked the fact that η(t) is also a function of θ itself. This could potentially jeopardize its stationary property. To address this issue, we refer to the slow dynamic (lazy dynamic) [27,28] of over-parameterized models under SGD optimization. This slow dynamic allows us to write the Taylor expansion (similar to what has been performed in neural tangent kernel theory [35], but with a different purpose) of the loss function around a microscopic state $\theta^{*}$, sampled from its current state $p_{t}\left(\theta \right)$:

(35) $$\phi_{\theta_{t}}\left(b_{t}\right)=\phi_{\theta^{*}}\left(b_{t}\right)+\left(\theta_{t}-\theta^{*}\right)\nabla_{\theta }\phi_{\theta^{*}}\left(b_{t}\right)$$

As a result, the gradient of the loss $\nabla_{\theta }\phi_{\theta_{t}}\left(b_{t}\right)=\nabla_{\theta }\phi_{\theta^{*}}\left(b_{t}\right)$, signifying an independent behavior from the specific value of the parameter $\theta_{t}$ at a given time *t*. We can extend this concept to the deterministic force $-\nabla_{\theta }U_{B}\left(\theta_{t}\right)=-\nabla_{\theta }U_{B}\left(\theta^{*}\right)=F\left(\theta^{*}\right)$, which indicates a conservative force in the lazy dynamics regime, denoted as $F(\theta^{*})$. This indicates a conservative force. The key point here is that the value of this force is independent of the exact microscopic state $\theta_{t}$, but rather on any typical sample $\theta^{*}$ from $\Theta_{t}$. In Appendix A, we illustrate how the condition of lazy dynamics leads to a thermodynamically reversible dynamic of the subsystem Θ.

### 6.1. Naive Parametric Reservoir

The stationary state of the subsystem Θ, under the dynamic of Equation (33) and satisfying the fluctuation-dissipation relation in Equation (34), corresponds to the thermal equilibrium state (the canonical state):

(36) $$p^{eq}=e^{-U_{B}\left(\theta \right)+F_{\Theta }}$$

where $F_{\Theta }:=-\log\left(\int d\theta e^{-U_{B}\left(\theta \right)}\right)$ is the free energy of the subsystem θ. Recall that the temperature was set to one. This state also satisfies the detailed balance condition, which defines the log ratio between the forward and backward transition probabilities (presented in Equation (16)) as follows:

(37) $$\log\frac{p(\theta_{t_{i}}|\theta_{t_{i-1}})}{p(\theta_{t_{i-1}|\theta_{t_{i}}})}=-\left(U_{B}\left(\theta_{t_{i}}\right)-U_{B}\left(\theta_{t_{i-1}}\right)\right)$$

The standard plot of the loss function versus the optimization steps in machine learning practice can help us to visualize the dynamics of the subsystem Θ. A rapid decline in the loss function signals a swift relaxation of the subsystem Θ to its equilibrium state. It is important to note that this *self-equilibrating property* is determined by the training dataset *B* through the definition of the potential function $U_{B}\left(\theta \right)$. These swift and self-equilibrating properties mirror the characteristics of a heat reservoir in thermodynamics [29].This observation supports the foundations on which we designated the subsystem Θ as the “parametric reservoir”. After a swift decline, a gradual reduction of the loss function can be a sign of a quasi-statistic process, where the subsystem Θ evolves from one equilibrium state to another. This can be due to the lazy dynamic condition, as discussed in Appendix A. Additionally, the requirement of a high heat capacity for the reservoir (dim(Θ)>>dim(X)), offers a thermodynamic justification for the use of over-parameterized models in machine learning.

### 6.2. Realistic Parametric Reservoir

We refer to the assumption of the parametric reservoir with an equilibrium state expressed in Equation (36) as the “naive assumption” due to several issues that were previously sidestepped. The first issue stems from the assumption that all components of the parameter vector θ are subject to the same temperature (i.e., $<\eta_{i}\left(t\right)\eta_{i}\left(t\right)>=\frac{2k_{B}T}{\mu }$ for all indexes *i*). In practice, we might find different values for the noise width, particularly with respect to different layers of a deep neural network. Furthermore, the weights or biases within a specific layer might experience different amounts of fluctuation. This scenario is entirely acceptable if we consider each group of parameters as a subsystem that contributes to the formation of the parametric reservoir. Consequently, each subsystem possesses different environmental temperatures and distinct stationary states. This observation may explain, in thermodynamic terms, why a deep neural network can offer a richer model. As it encompasses multiple heat reservoirs at varying temperatures, it presents a perfect paradigm for the emergence of non-equilibrium thermodynamic properties.

Second, the fluctuation term η may exhibit an autocorrelation property that characterizes colored noise, as presented in [36]. While this introduces additional richness to the problem, potentially displaying non-Markovian properties, it does not impede us from deriving the equilibrium state, as demonstrated in [37].

We also overlooked the irregular behavior of the loss function, such as spikes or step-like patterns. These irregularities are considered abnormal as we typically expect the loss function to exhibit a monotonous decline, but in practice, such behaviors are quite common. These anomalies may be associated with a more intricate process experienced by the reservoir, such as a phase transition or a shock. Nevertheless, we can still uphold the basic parametric reservoir assumption during the time intervals between these irregular behaviors.

The mentioned issues are attributed to a richer and more complex dynamic of the subsystem Θ and do not fundamentally contradict the potential role of subsystem Θ as a reservoir. Examples of these richer dynamics can be found in a recent study [38], which shows the limitation of the Langevin formulation of the SGD, and Ref. [39], which investigates the exotic non-equilibrium characteristic of parameters’ dynamics under SGD optimization.

Before closing this section, it is worth mentioning that the experimental results presented in Figure 3 support the assumption of a low-variance condition for the stochastic dynamics of the subsystem Θ. For instance, panel (a) shows that even in the high noise regime ($|b_{t}|=1$), the dynamics of the parameters remained confined to a small region across the ensemble. Furthermore, panel (b) demonstrates the low-variance characteristics of the model’s performance accuracy. Finally, the large magnitude of the deterministic force (dashed line in panel (c)) to the random force is evidence of low-variance dynamics.

## 7. Discussion

In this study, we delved into the thermodynamic aspects of machine learning algorithms. Our approach involved first formulating the learning problem as the time evolution of a PPM. Consequently, the learning process naturally emerged as a thermodynamic process. This process is driven by the work of the optimizer, which can be considered a thermodynamic work since parameter optimization constantly changes the system’s energy landscape through $\phi_{\theta }\left(x\right)$. The optimizer action is fueled by the input trajectory and a series of samples drawn from the ground truth system. The work and heat exchange of the subsystem *X* can be computed practically along the learning trajectory T as outlined below:

(38) $$\begin{array}{cc} W_{X}\left(\theta_{n}\right) & =\sum_{t=1}^{n}<\phi_{\theta_{t}}\left(x\right){>}_{p(x|\theta_{t-1})}-<\phi_{\theta_{t-1}}\left(x\right){>}_{p(x|\theta_{t-1})} \end{array}$$

(39) $$\begin{array}{cc} Q_{X}\left(\theta_{n}\right) & =\sum_{t=1}^{n}<\phi_{\theta_{t}}\left(x\right){>}_{p(x|\theta_{t})}-<\phi_{\theta_{t}}\left(x\right){>}_{p(x|\theta_{t-1})} \end{array}$$

We use the term “practically” because when running a machine learning algorithm, we have access to the function $\phi_{\theta }\left(x\right)$ at each training instance. We also note that these quantities are conditioned on a specific parameter’s trajectory as a result of working in the conditional view. Finally, the learning process can be summarized as follows: *The model learns by dissipating heat, and the dissipated heat increases the entropy of the parameters, which act as the heat reservoir (a memory space) for learned information.* This means the learning process must be irreversible, as this is the only way to increase the mutual information between the two subsystem *X* and Θ [10].

It is important to note that despite the wealth of research highlighting the significance of information content in parameters [21,22,25], calculating these quantities remains difficult due to the lack of access to the parameter distribution. In contrast, the thermodynamic approach computes the information-theoretic metrics indirectly as the heat and work of the process. Moreover, the mysterious success of over-parametrized models can be explained within the thermodynamic framework, where over-parameterization plays a crucial role in allowing the parameter subsystem to function as a heat reservoir.

At the same time, we are aware of the strong assumptions made during this study. Addressing each of these assumptions or providing justifications for them represents a direction for future research. For instance, we assumed slow dynamics for over-parameterized models using the SGD optimizer. This formed the basis for treating the parameters’ degrees of freedom as an ideal heat reservoir, evolving in a thermodynamically reversible manner. Breaking this assumption leads to entropy production of parameter subsystem, changing contribution of entropy production to accumulation of L-info.

We also sidestepped the role of changes in the marginal entropy of the model’s subsystem $\Delta_{t_{n}}S_{X}\left(t\right)$. This term can be estimated by computing the entropy of the empirical distribution of generated samples. For a model initialized randomly, this term is always negative, as the initial model produces uncorrelated patterns with maximum entropy. Then, the negative value of this term must converge when the entropy of the generated patterns reaches the entropy of the training dataset, or a representation of the training dataset that is most relevant to the learning objective. However, if we look at Equation (25) as an optimization objective to maximize the L-info, then an increase in the model’s generated samples $S_{X}\left(t\right)$ is favorable. This might act as a regularization term to improve the generalization power of the model by forcing it to avoid easy replication of the dataset.

## Figures and Tables

**Table 1:** A list of notations used in this paper.

$\Delta_{t_{n}}f\left(t\right):=f\left(t_{n}\right)-f\left(0\right)$	Change over the interval $\left[0,t_{n}\right]$
$<f\left(x\right){>}_{p(x)}:=\int dx\;p\left(x\right)\;f\left(x\right)$	Average over p(x)
$s_{X}\left(t\right):=s\left[p_{t}\left(x\right)\right]:=-\ln p_{t}\left(x\right)$	Surprisal of $p_{t}\left(x\right)$
$S_{X}\left(t\right):=S\left[p_{t}\left(x\right)\right]:=<-\ln p_{t}\left(x\right){>}_{p_{t}\left(x\right)}$	Shannon entropy of $p_{t}\left(x\right)$
$I_{X;\Theta }\left(t\right):=I\left[X_{t};\Theta_{t}\right]:=S_{X}\left(t\right)-S_{X|\Theta }\left(t\right)$	Mutual information between *X* and Θ at time t

## Data Availability

This is a more theoretical work, data sharing is not applicable to this article.

## Appendix A. Reversibility under the Lazy Dynamic Regime

In this appendix, we establish the thermodynamic reversibility of parameter evaluation as a consequence of training an over-parameterized model with lazy dynamics. The forward action of the optimizer can be summarized as follows: $b_{n}\to \theta_{n}$, where the optimizer samples an independent and identically distributed trajectory of inputs from the training dataset, $b_{n}=\left\{b_{t_{1}},b_{t_{2}},\cdots ,b_{t_{n}}\right\}$, to generate a trajectory of the updated parameters, $b_{n}=\left\{\theta_{0},\theta_{t_{1}},\cdots ,\theta_{t_{n}}\right\}$.

The backward (time reversal) action of the optimizer is defined as ${\tilde{b}}_{n}\to \theta_{n}^{†}$, where ${\tilde{b}}_{n}=\left\{b_{t_{n}},b_{t_{n-1}},\cdots ,b_{t_{1}}\right\}$ represents the time reversal of the input trajectory and the gradient descent is reversed to a gradient ascent, resulting in a new parameter trajectory $\theta_{n}^{†}$.

In general, the backward action of the SGD does not yield the time reversal of the forward parameter trajectory:

$$\begin{array}{c} \theta_{n}^{†}\neq {\tilde{\theta }}_{n}=\left\{\theta_{t_{n}},\theta_{t_{n-1}},\cdots ,\theta_{t_{0}}\right\} \end{array}$$

To illustrate this, let us examine a single forward and backward action of the optimizer:

$$\begin{array}{cc} \theta_{t+1} & =\theta_{t}-r\nabla_{\theta }\phi_{\theta }\left(b_{t}\right){|}_{\theta =\theta_{i}}\;\;\;\;\;\;\;\;\;\;\;\;\;(\mathrm{Forward}\;\mathrm{step})\\ \theta_{t}^{†} & =\theta_{t+1}+r\nabla_{\theta }\phi_{\theta }\left(b_{t}\right){|}_{\theta =\theta_{i+1}}\;\;\;\;\;\;\;\;(\mathrm{Backward}\;\mathrm{step}) \end{array}$$

This discrepancy arises due to the gradient step’s dependence on the current values of the parameters in both the forward and backward optimizations (i.e., $\nabla_{\theta }\phi_{\theta }\left(b_{t}\right){{|}_{\theta =\theta_{i}}\neq \nabla_{\theta }\phi_{\theta }\left(b_{t}\right)|}_{\theta =\theta_{i+1}}$).

However, the key observation here is that under the lazy dynamic regime (as described in Equation (35)), this dependency vanishes, and both forward and backward gradient step can be written as $\nabla_{\theta }\phi_{\theta }\left(b_{t}\right){|}_{\theta =\theta *}$, where $\theta^{*}$ is a typical sample from the stationary state (or slowly varying state) of parameters. Under such conditions, the backward action of the SGD (running the learning protocol backward) results in a time reversal of the parameters’ trajectory, namely $\theta_{n}^{†}={\tilde{\theta }}_{n}$, signifying the thermodynamic reversibility of the parameters’ subsystem under lazy dynamic conditions. Thus, the lazy dynamics lead to a quasi-static evolution of the parameter subsystem, meaning that the subsystem Θ itself does not contribute to entropy production and acts as an ideal heat reservoir. See Ref. [40] for a detailed discussion on thermodynamic reversibility.

## References

[1] Landauer R. (1961). Irreversibility and heat generation in the computing process. IBM J. Res. Dev..

[2] Szilard L. (1929). On the decrease of entropy in a thermodynamic system by the intervention of intelligent beings. Z. Phys..

[3] Bennett C.H. (1982). The thermodynamics of computation—A review. Int. J. Theor. Phys..

[4] Nielsen M.A., Chuang I.L. (2010). Quantum Computation and Quantum Information: 10th Anniversary Edition.

[5] Almheiri A., Hartman T., Maldacena J., Shaghoulian E., Tajdini A. (2021). The entropy of hawking radiation. Rev. Mod. Phys..

[6] Parrondo T.S.J., Horowitz J. (2015). Thermodynamics of information. Nat. Phys..

[7] Peliti L., Pigolotti S. (2021). Stochastic Thermodynamics: An Introduction.

[8] Still S., Sivak D.A., Bell A.J., Crooks G.E. (2012). Thermodynamics of prediction. Phys. Rev. Lett..

[9] Sagawa T., Ueda M. (2012). Fluctuation theorem with information exchange: Role of correlations in stochastic thermodynamics. Phys. Rev. Lett..

[10] Esposito M., Lindenberg K., den Broeck C.V. (2010). Entropy production as correlation between system and reservoir. New J. Phys..

[11] Song Y., Kingma D.P. (2021). How to train your energy-based models. arXiv.

[12] Kingma D.P., Welling M. (2013). Auto-encoding variational bayes. arXiv.

[13] Jeon H.J., Zhu Y., Roy B.V. (2022). An information-theoretic framework for supervised learning. arXiv.

[14] Yi J., Zhang Q., Chen Z., Liu Q., Shao W. (2022). Mutual information learned classifiers: An information-theoretic viewpoint of training deep learning classification systems. arXiv.

[15] Shwartz-Ziv R., LeCun Y. (2023). To compress or not to compress- self-supervised learning and information theory: A review. arXiv.

[16] Yu S., Giraldo L.G.S., Príncipe J.C. Information-theoretic methods in deep neural networks: Recent advances and emerging opportunities. Proceedings of the Thirtieth International Joint Conference on Artificial Intelligence.

[17] Geiger B.C. (2021). On information plane analyses of neural network classifiers—A review. arXiv.

[18] Achille A., Paolini G., Soatto S. (2019). Where is the information in a deep neural network?. arXiv.

[19] Shwartz-Ziv R., Tishby N. (2017). Opening the black box of deep neural networks via information. arXiv.

[20] Andrew M., Bansal Y., Dapello J., Advani M., Kolchinsky A., Tracey B.D., Cox D.D. (2018). On the information bottleneck theory of deep learning. J. Stat. Mech. Theory Exp..

[21] Hinton G.E., Camp D.V. Keeping the neural networks simple by minimizing the description length of the weights. Proceedings of the Sixth Annual Conference on Computational Learning Theory.

[22] Achille A., Soatto S. (2018). Emergence of invariance and disentanglement in deep representations. J. Mach. Learn. Res..

[23] Cover T.M., Thomas J.A. (1991). Elements of Information Theory.

[24] Rissanen J. (1986). Stochastic complexity and modeling. Ann. Stat..

[25] Bu Y., Zou S., Veeravalli V.V. (2020). Tightening mutual information-based bounds on generalization error. IEEE J. Sel. Areas Inf. Theory.

[26] den Broeck C.V., Esposito M. (2015). Ensemble and trajectory thermodynamics: A brief introduction. Phys. A Stat. Mech. Its Appl..

[27] Du S.S., Zhai X., Poczos B., Singh A. (2018). Gradient descent provably optimizes over-parameterized neural networks. arXiv.

[28] Li Y., Liang Y. (2018). Learning overparameterized neural networks via stochastic gradient descent on structured data. Adv. Neural Inf. Process. Syst..

[29] Deffner S., Jarzynski C. (2013). Information processing and the second law of thermodynamics: An inclusive, hamiltonian approach. Phys. Rev. X.

[30] Du Y., Mordatch I. (2019). Implicit generation and generalization in energy-based models. arXiv.

[31] Maes C. (2021). Local Detailed Balance.

[32] Rao R., Esposito M. (2018). Detailed fluctuation theorems: A unifying perspective. Entropy.

[33] Zwanzig R. (2001). Nonequilibrium Statistical Mechanics.

[34] Wei M., Schwab D.J. (2019). How noise affects the hessian spectrum in overparameterized neural networks. arXiv.

[35] Jacot A., Gabriel F., Hongler C. (2018). Neural tangent kernel: Convergence and generalization in neural networks. Adv. Neural Inf. Process. Syst..

[36] Kühn M., Rosenow B. (2023). Correlated noise in epoch-based stochastic gradient descent: Implications for weight variances. arXiv.

[37] Ceriotti M., Bussi G., Parrinello M. (2009). Langevin equation with colored noise for constant-temperature molecular dynamics simulations. Phys. Rev. Lett..

[38] Ziyin L., Li H., Ueda M. (2023). Law of balance and stationary distribution of stochastic gradient descent. arXiv.

[39] Adhikari S., Kabakçıoğlu A., Strang A., Yuret D., Hinczewski M. (2023). Machine learning in and out of equilibrium. arXiv.

[40] Sagawa T. (2014). Thermodynamic and logical reversibilities revisited. J. Stat. Mech. Theory Exp..

